# Recent Advances in Nanoporous Membranes for Water Purification

**DOI:** 10.3390/nano8020065

**Published:** 2018-01-25

**Authors:** Zhuqing Wang, Aiguo Wu, Lucio Colombi Ciacchi, Gang Wei

**Affiliations:** 1 AnHui Province Key Laboratory of Optoelectronic and Magnetism Functional Materials, Anqing Normal University, Anqing 246011, China; wangzhuqinggg@163.com; 2 Hybrid Materials Interfaces Group, Faculty of Production Engineering and Center for Environmental Research and Sustainable technology (UFT), University of Bremen, D-28359 Bremen, Germany; colombi@uni-bremen.de; 3 CAS Key Laboratory of Magnetic Materials and Devices, Key Laboratory of Additive Manufacturing Materials of Zhejiang Province, Division of Functional Materials and Nanodevices, Ningbo Institute of Materials Technology and Engineering, Chinese Academy of Sciences, Ningbo 315201, China; aiguo@nimte.ac.cn; 4 MAPEX Center for Materials and Processes, University of Bremen, Am Fallturm 1, D-28359 Bremen, Germany

**Keywords:** nanoporous membrane, two-dimensional materials, water purification, water pollutants, purification mechanism, graphene, fabrication

## Abstract

Nanoporous materials exhibit wide applications in the fields of electrocatalysis, nanodevice fabrication, energy, and environmental science, as well as analytical science. In this review, we present a summary of recent studies on nanoporous membranes for water purification application. The types and fabrication strategies of various nanoporous membranes are first introduced, and then the fabricated nanoporous membranes for removing various water pollutants, such as salt, metallic ions, anions, nanoparticles, organic chemicals, and biological substrates, are demonstrated and discussed. This work will be valuable for readers to understand the design and fabrication of various nanoporous membranes, and their potential purification mechanisms towards different water pollutants. In addition, it will be helpful for developing new nanoporous materials for quick, economic, and high-performance water purification.

## 1. Introduction

Water is the source of life, and one of the most important material resources for human survival and development. Although 71% of the earth’s surface is covered with water, freshwater resources that can be directly used by humans, such as river water, freshwater lakes and shallow groundwater, account for only 0.03% of the total water amount. Moreover, with the rapid development of industries and increasing human activities, such as metal plating, fertilizers, tanneries, mining, paper, batteries, pesticides, and etc., many harmful inorganic and organic pollutants are released into water, which seriously endangers the freshwater resource and ecological environment [1,2]. 

Heavy metallic ions in water are difficult to biodegrade, and they can enter the human body through the food chain, causing a series of irreversible physiological diseases. For example, mercury ions can damage the central nervous system, leading to headaches, stomatitis, and gastroenteritis [3]. Lead ions can cause an inadequate supply of nutrients and oxygen, resulting in brain tissue damage [4]. Especially for children in their growth and development stages, excessive lead ions in their bodies would lead to developmental delay, loss of appetite, and hearing impairment. Cadmium ions can replace calcium ions in the bones, hinder the normal deposition of calcium in the bone, and result in cartilage disease [5]. Excess arsenic in the human body can interfere with the normal metabolism of cells, causing cell lesions and leading to organ damage. Organic pollutants in water such as pesticides, fertilizes, hydrocarbons, phenols, plasticizers, biphenyls, detergents, oils, greases, and pharmaceuticals are mainly derived from domestic sewage, agriculture, food, and paper industry. These organic pollutants need to consume a lot of oxygen in the process of oxidative decomposition, which will reduce the amount of dissolved oxygen in water, thereby endangering aquatic organisms and ecosystems [6]. In addition, some harmful microbes in water such as algae, fungi, planktons, virus, bacteria, and amoebas are responsible for causing illnesses called waterborne diseases [7]. Therefore, developing efficient water purification materials and green wastewater treatment methods are urgent issues that need to be solved by governments and scientists.

Currently, many technologies such as chemical precipitation [8], adsorption [9], ion exchange [10], membrane filtration [11], electrochemistry [12], phytoremediation [13], and others [14] have been widely employed to purify wastewater. Due to its advantages for operation at room temperature, simple operation processes, low energy consumption, high efficiency, and small investment, the membrane filtration technique has drawn more attention than other treatment technologies for water purification. Microporous membranes with a pore size about 0.1–5 µm can only be used for filtering particles with 1–10 µm, which limits their applications in water purification. Meanwhile, nanoporous membranes exhibited high performance for water purification. They can filter most of the pollutants (1–10 nm) such as metallic ions, organic molecules, salts, and microbes from wastewater [15,16]. To design high performance nanoporous membranes for water purification, many kind of inorganic, organic, and inorganic-organic hybrid materials have been utilized [17,18,19]. For instance, Surwade et al. demonstrated the water desalination by using nanoporous single-layer graphene [20]; Zhang et al. reported an ultrathin nanoporous membrane formed with sub-10 nm diameter cellulose nanofibers, which exhibited fast permeation of water and organics [21]; Bolisetty and Mezzenga designed the amyloid-carbon hybrid membranes for wastewater purification [22].

Previously, several important reviews on the fabrication of membranes with various techniques and materials have been reported [17,18,23,24,25,26,27]. For example, Lee et al. summarized recent studies on the design, development, and applications of membrane materials for water purification [18]. Lalia and co-workers elucidated the relationship between the structure, properties and performance of membranes by comparing various membrane fabrication techniques [24]. Recently, a few reviews on the fabrication of membranes by using graphene and other two-dimensional (2D) nanomaterials for water treatment have been released [25,26]. Due to the growing importance of nanoporous structures of membranes for water treatments, recent advances in their design, fabrication, and water purification application need to be summarized.

Therefore, in this review we focus on nano-filtration (NF), ultra-filtration (UF), and reverse osmosis (RO) of wastewater by nanoporous membranes. We first introduce the synthesis strategies of nanoporous membranes, and then demonstrate the applications of different types of nanoporous membranes in wastewater purification. The fabricated nanoporous membranes for removing various water pollutants such as salt, metallic ions, anions, nanoparticles, organic chemicals, and biological contaminants are presented and discussed. Finally, we analyze and discuss the advantages and disadvantages of porous membranes in the water filtering process, and look ahead to the prospects and development of nanoporous membranes in the field of water treatment.

## 2. Types and Fabrication Methods of Nanoporous Membranes

### 2.1. Types of Nanoporous Membranes

Nanoporous membrane for water purification can generally be divided into three types based on their material composition: inorganic, organic, and inorganic-organic hybrid membranes. Inorganic membranes are mainly made of ceramics (Al_2_O_3_, TiO_2_, ZrO_2_, SiO_2_, TiO_2_-SiO_2_, TiO_2_-ZrO_2_, Al_2_O_3_-SiC) [28,29,30,31,32,33], graphene [34,35,36], and carbon nanotubes (CNTs) [37,38]. Organic membranes are mainly made of polymeric materials such as polyvinyl alcohol (PVA), polyimide (PI), polypropylene (PP), polyethersulfone (PES), cellulose acetate (CA), cellulose nitrates, polysulfone (PSU), polyvinylidene fluoride (PVDF), polyacrylonitrile (PAN), polytetrafluoroethylene (PTFE), and biomacromolecules [39,40,41,42,43,44,45]. Inorganic-organic hybrid membranes are usually made by introducing inorganic materials (metals, metal oxide, or carbon-based materials) into a polymeric matrix system [46,47,48,49,50,51,52]. 

### 2.2. Fabrication Methods of Nanoporous Membranes

**Figure 1:**
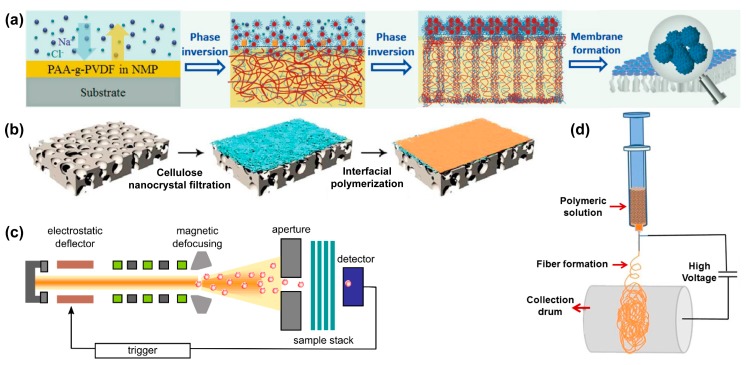
Fabrication strategies of various nanoporous membranes: (**a**) Formation of a superoleophobic PAA-g-PDVF membrane by a salt-induced phase-inversion process; (**b**) Preparation of triple-layered thin film composite (TFC) nano-filtration membrane by interfacial polymerization; (**c**) Schematic of single-ion irradiation setup; and (**d**) Schematic of electrospinning. Picture (**a**) is reprinted with permission from Ref. [57]. Copyright Wiley-VCH (Weinheim, Germany), 2016. Picture (**b**) is reprinted with permission from Ref. [58]. Copyright Royal Society of Chemistry, 2017. Picture (**c**) is reprinted with permission from Ref. [59]. Copyright Beilstein-Institut, 2012. Picture (**d**) is reprinted with permission from Ref. [54]. Copyright Elsevier, 2015.

Currently, the most common techniques for fabricating the above three types of nanoporous membranes include phase inversion, interfacial polymerization, track-etching, and electrospinning [24,53,54,55,56], as shown in Figure 1. In this section, the basic mechanisms for the fabrication of nanoporous membranes and corresponding typical cases are discussed briefly.

#### 2.2.1. Phase Inversion

Phase inversion is a stratification process that converts a homogeneous solution into a solid state in a controlled manner [44,57,60]. The transformation can be accomplished by immersion precipitation, thermo-induced phase separation, vapor-induced phase separation, and evaporation-induced phase separation. Among these techniques, immersion precipitation and thermo-induced phase separation are the most common method for the fabrication of NF, UF, and RO membranes [61,62,63]. The morphology of membrane can be controlled by using solvents with different boiling points and porous shape of the substrate. For example, Zhang and co-workers fabricated a superoleophobic poly(acrylic acid)-grafted PVDF (PAA-g-PDVF) filtration membrane by salt-induced phase-inversion approach for treating emulsified oil/water mixtures (Figure 1a) [57].

#### 2.2.2. Interfacial Polymerization

The interfacial polymerization method has been previously used to fabricate RO and NF membranes [58]. This technique is based on a polycondensation reaction between two monomers (such as polyamines and polyacyl chlorides) dissolved in immiscible solvents, one of which, the aqueous polyamine solution, initially impregnates the substrate [64,65]. An ultra-thin film (from 10 nm to several μm thick) could be quickly formed at the polymer-substrate interface and attached to the substrate. The structural morphology of the membranes created with this method can be controlled by concentration of monomers, reaction time, solvent type, and post-treatment conditions [66,67]. For instance, Wang et al. prepared a triple-layered composite NF membrane by the interfacial polymerization of diamine and acyl chloride on a cellulose nanocrystal interlayer for water desalination (Figure 1b) [58]. Yin and co-workers prepared nanocomposite membranes by using MCM-41 silica nanoparticles and graphene oxide (GO)-enhanced PA thin films via the interfacial polymerization method [68,69], and both nanoporous membranes exhibited high performances for water purification.

#### 2.2.3. Track-Etching

In the track-etching process, non-porous polymer membranes are irradiated with high energy heavy ions, resulting in a linearly damaged track across the irradiated polymer membrane to form nanopores [59], as indicated in Figure 1c. The track-etching technique can precisely control the pore size distribution of the membrane, and the pore size and pore density can be adjusted in the range of a few nm to tens of μm and 1 to 10^10^ cm^−2^, respectively [70]. Previously, DesOrmeaux et al. fabricated a nanoporous silicon nitride membrane from porous nanocrystalline silicon template through the track-etching process [71].

#### 2.2.4. Electrospinning

Electrospinning is a simple and effective technique developed 20 years ago to fabricate nanofibrous membrane materials [72,73,74,75,76,77]. As shown in Figure 1d, a high voltage is applied between the polymer solution droplet and the collector. When the applied voltage is large enough to overcome the surface tension of the droplets, a charged liquid jet is formed [54]. The porosity, pore size distribution, aspect ratios, and morphology of the nanofibrous membranes prepared by electrospinning can be controlled by adjusting the solution viscosity, applied electric potential, environmental conditions, and the flow of the solution [78]. For instance, by using electrospinning, Feng et al. prepared a PVDF nanofibrous membrane for removing NaCl [79], and in another study Ren et al. prepared *Ti*_3_*C*_2_*T_x_* mxene membranes for sieving cations and dyes from wastewater [80].

## 3. Applications for Water Purification

According to the properties of the target water pollutes, we classify them into three categories, namely inorganic pollutants, organic pollutants, and biological pollutants. In this section we introduce and discuss the applications of corresponding nanoporous membranes for purifying these three types of pollutants. 

### 3.1. Removal of Inorganic Contaminants

#### 3.1.1. Removing Cations

Due to their excellent permeability, and mechanical and chemical stabilities, polymer materials have been widely used for producing water purification membranes or as supporting substrates for fabricating hybrid membranes. For example, Feng and co-workers designed and prepared a PVDF nanofibrous membrane via electrospinning for water desalination [79]. The prepared membrane produced potable water successfully (with a concentration of NaCl lower than 280 ppm) starting from saline water with 6 wt % NaCl, which is comparable to the performance of commercial membranes. The membrane flux reached 5–28 kg m^−2^ h^−1^ at the temperature ranging from 15 °C to 83 °C. Moreover, the water purification performance of this membrane remained stable after several days of using. Sun et al. developed a NF membrane by immobilizing AquaporinZ-reconstitued liposomes onto a PDA-coated microporous membrane [81]. The obtained membrane retained 66.2% of NaCl and 88.1% of MgCl_2_, respectively. Because AquaporinZ is a water-channel protein and have extraordinary water permeability and selectivity, the membranes functionalized with an AquaporinZ-to-lipid weight ratio of 1:100 increased the water flux by 65% when compared with the membranes without AquaporinZ incorporation. In another study, Tijing and co-workers fabricated a superhydrophobic PVDF-co-hexafluoropropylene nanofiber membrane containing CNTs by one-step electrospinning method [82]. The mechanical and hydrophobic properties of this membrane could be controlled by adjusting the concentration of the incorporation CNTs. Incorporation of 5 wt % CNTs to the membrane led to highest water flux (24–29.5 L m^−2^) and more than 99.99% salt retention under an external pressure (≥liquid entry pressure) of 99 kPa. 

**Figure 2:**
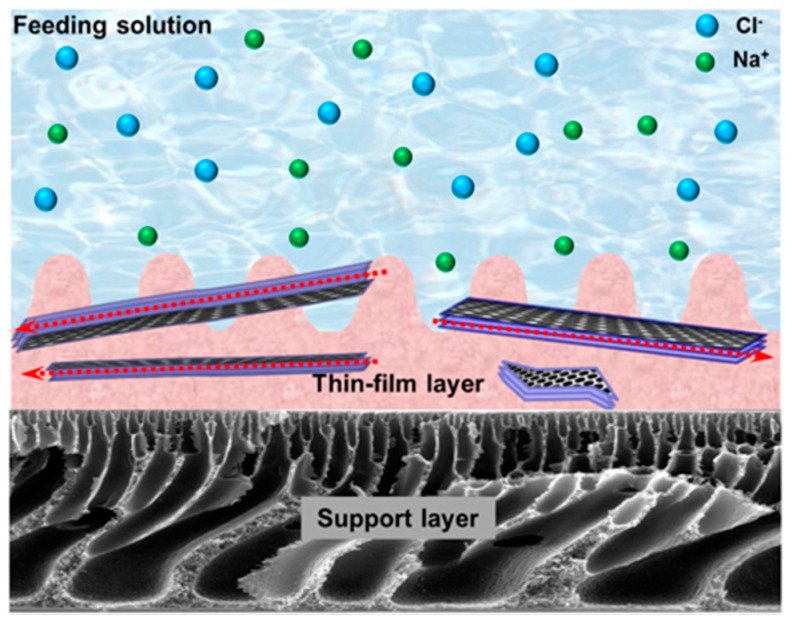
Schematic illustration of the hypothesized mechanism of graphene oxide (GO) thin-film nanocomposite membrane. Reprinted with permission from Ref. [69]. Copyright Elsevier, 2016.

Inorganic nanoporous membranes can also be used for removing cations from wastewater. For example, Liu et al. prepared a continuous zirconium(IV)-based metal-organic framework (Zr-MOF) membranes on porous, hollow ceramic fibers by using an in situ solvothermal synthesis method for water desalination. The obtained Zr-MOF membrane (i.e., UiO-66) exhibited high multivalent ion retention (99.3% for Al^3+^, 98.0% for Mg^2+^, and 86.3% for Ca^2+^) on the basis of size-exclusion mechanism. It showed good permeability (0.28 L m^−2^ h^−1^ bar^−1^) and good chemical stability in various saline tests [83]. In another study, Yin and co-workers fabricated a thin-film nanocomposite (TFN) membrane containing porous MCM-41 silica nanoparticles via the in-situ interfacial polymerization for water purification [68]. The prepared membrane showed high retentions of NaCl and Na_2_SO_4_ (97.9% ± 0.3% for NaCl, and 98.5% ± 0.2% for Na_2_SO_4_). The incorporated MCM-41 nanoparticles not only increased the hydrophilicity, roughness, and zeta potential of the TFN membrane, but also increased the permeate water flux from 28.5 ± 1.0 to 46.6 ± 1.1 L m^−2^ h^−1^. To improve the water flux, the authors further replaced the MCM-41 silica nanoparticles with GO to fabricate a membrane [69]. As shown in Figure 2, the interlayer spacing of GO nanosheets could serve as water channel and promote water permeability. The water flux of GO-enhanced polyamide TFN membrane reached up to 59.4 ± 0.4 L m^−2^ h^−1^, and the rejection coefficients of NaCl and Na_2_SO_4_ were 93.8% ± 0.6% and 97.3% ± 0.3%, respectively. 

**Figure 3:**
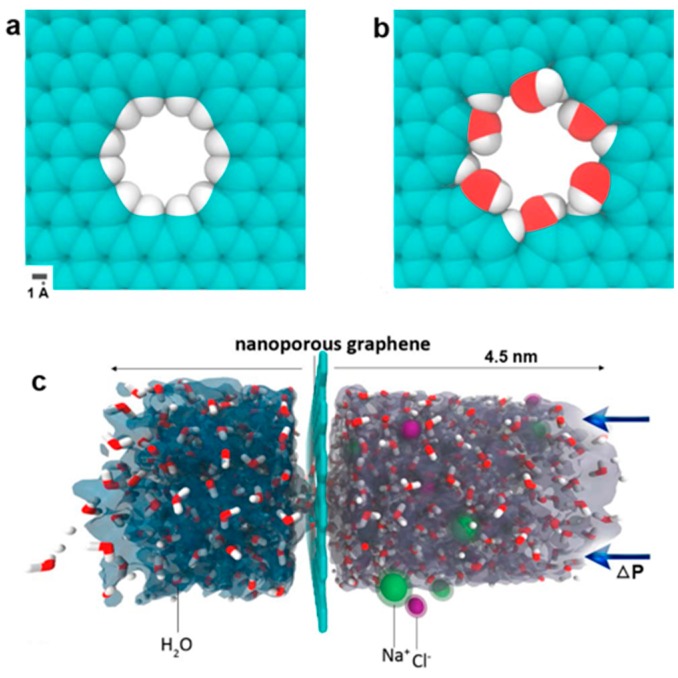
(**a**) Hydrogenated and (**b**) hydroxylated graphene pores; and (**c**) side view of computational system investigated. Figure reprinted with permission from Ref. [35]. Copyright American Chemical Society, 2012.

Two-dimensional graphene materials are excellent building blocks for fabricating nanoporous membranes for water purification. For instance, Chen and co-workers discovered that the spacing of GO membranes (GOMs) can be precisely controlled by added cations due to the strong cation-π interactions between hydrated cations and aromatic rings of graphene. The membrane spacing controlled by one type of cation can efficiently and selectively exclude other cations that have larger hydrated volumes. For instance, K^+^-controlled GOMs could efficiently reject Mg^2+^, Ca^2+^, Na^+^ and Li^+^, and showed stable performance for over 24 h with a water flux of 0.36 L m^−2^ h^−1^ [84]. By using classical molecular dynamics simulations, David et al. reported that nanoscale pores in single-layer freestanding graphene can efficiently filter NaCl salt from water [35]. The simulation results indicated that the desalination performance is very sensitive to the pore size and pore chemistry of a graphene membrane (Figure 3). Later, Sumedh et al. created tunable nanoscale pores on a monolayer graphene membrane by using an oxygen plasma etching method. The obtained membrane showed a rejection rate of nearly 100% for salt ions (K^+^, Na^+^, Li^+^, Cl^−^), and up to 10^6^ g m^−2^ s^−1^ of water fluxes at 40 °C by using pressure difference as a driving force [20]. Although scaling up of these monolayer membranes remains a significant challenge, the effectiveness and potential of nanoporous graphene for desalination applications are very fascinating.

Hu et al. assembled GO nanosheets into a GO membrane layer-by-layer method on a polydopamine-coated polysulfone support by using 1,3,5-benzenetricarbonyl trichloride as cross-linker [85]. The obtained GO membrane showed relatively low rejection (6–46%) of salt cations, but the water flux could reach 27.6 L m^−2^ h^−1^ bar^−1^, which is much higher than that of most commercial NF membranes. Later, Wang and co-workers fabricated a GO NF membrane on a highly porous polyacrylonitrile nanofibrous mat (GO@PAN) by a simple vacuum suction method [86]. The thickness of GO layer can be controlled by manipulating the concentration of GO solution. The obtained GO@PAN membrane exhibited high water flux (8.2 L m^−2^ h^−1^ bar^−1^) and nearly 100% rejection of Congo red and 56.7% rejection of Na_2_SO_4_. Liu et al. fabricated freestanding ultrathin reduced graphene oxide (rGO) membranes by hydriodic acid vapor and water-assisted delamination [87]. Thanks to the smaller nanochannels between rGO nanosheets, the achieved rGO membrane showed nearly 100% rejection of Cu^2+^, Na^+^, and orange 7. Moreover, the water flux of the fabricated rGO membrane reached 12.0 L m^−2^ h^−1^ bar^−1^. In another study, Akbari and co-workers developed large-area GO-based NF membranes by shear alignment of discotic nematic liquid crystals of GO [88]. The highly ordered graphene sheets in the GO-based membrane not only enhanced the water permeability (71 ± 5 L m^−2^ h^−1^ bar^−1^), but also could sieve ˃90% of organic probe molecules (hydrated radius above 5 Å) and 30–40% of salt cations.

**Figure 4:**
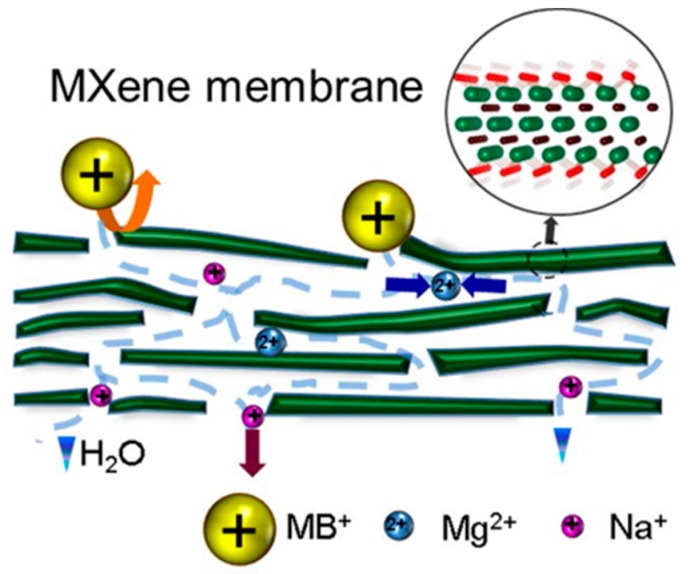
Mechanism of charge- and size-selective ion sieving through MXene membrane. Figure reprinted with permission from Ref. [80]. Copyright American Chemical Society, 2015.

2D graphene-like materials show similar separation ability as graphene-based membranes. Previously, Ren et al. assembled 2D Ti_3_C_2_T_x_ (MXene) into nm-thin membrane for charge- and size- selective rejection of ions and molecules [80]. The prepared MXene membrane showed ultrafast water flux of 37.4 L m^−2^ h^−1^ bar^−1^, and high selectivity toward single-, double- and triple-charged metal cations and dye cations with different sizes. As shown in Figure 4, cations (such as MB^+^, Ca^2+^) with a larger hydration radius and charge smaller than the interlayer spacing of MXene (6 Å) showed an order of magnitude slower permeation compared to single-charged cations (such as Na^+^). 

#### 3.1.2. Removing Anions

**Figure 5:**
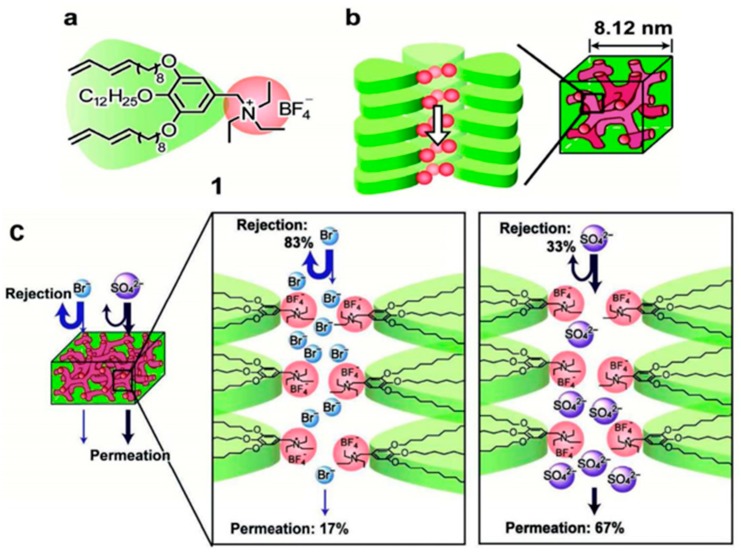
(**a**) Chemical structure of the liquid-crystal molecule 1; (**b**) Self-assembled bicontinous cubic (Cub_bi_) structure forming ionic nanochannels of 1; (**c**) Schematic representation of selective rejection of anions through the Cub_bi_ membranes. Figures reprinted with permission from Ref. [89]. Copyright Wiley-VCH, 2012.

Henmi et al. synthesized a bicontinous cubic (Cub_bi_) membrane through the self-assembly of thermotropic liquid-crystal (LC) molecules [89], as shown in Figure 5. The obtained Cub_bi_ membrane has self-organized pores with average size of 0.6 nm, and exhibits anions rejection properties and unique ion selectivity. The experimental results indicated that the Cub_bi_ membrane can reject 83% of Br^−^, 59% of Cl^−^, 33% of SO_4_^2−^, and 81% of NO_3_^−^, respectively. This self-organized nanostructured RO membranes have great potential for removing a lot of solutes and afford high-quality potable water, agriculture and industrial water. Mezzenga and co-workers prepared a hybrid composite membrane by incorporating β-lactoglobulin, amyloid fibrils and activated carbon via vacuum filtration. The obtained membrane could remove more than 99% of ${\mathrm{AsO}}_{4}^{3-}$ and ${\mathrm{AsO}}_{3}^{3-}$ from arsenic-contaminated water via strong supramolecular metal-ligand interactions. Moreover, this membrane could be reused for several cycles without any efficiency drop [90].

When compared with the cation-rejection membrane, it can be concluded that the anion-rejection membrane repel anions mainly by electrostatic interaction or channel surface coordination groups (such as sulfhydryl group). However, the cation-rejection membrane repel cations mainly by the pore size of the membrane.

#### 3.1.3. Removing Nanoparticles

Nanoparticles can easily enter human body through the food chain (such as drinking water) due to their small size, and this can result in cellular oxidative stress and inflammation. Therefore, the removal of nanoparticles from water is of great significance. Previously, DesOrmeaux and co-workers fabricated a nanoporous silicon nitride (NPN) membrane by using porous nanocrystalline silicon as a reactive ion etching layer for removing gold (Au) nanoparticles from water [71]. The pore size of the NPN membrane could be adjusted from 40 to 80 nm by modifying the mask layer and controlling the reactive ion etching conditions. The obtained membrane could remove more than 80% of Au nanoparticles (diameter ≥ 60 nm) from water. Zhang et al. reported a versatile approach to fabricate ultrathin nanoporous membranes with 7.5 nm diameter cellulose nanofibers by using a freeze-extraction technique [21]. The thickness of obtained membrane could be controlled to as thin as to 23 nm, with pore sizes ranging from 2.3 nm to 12 nm. The experimental results indicated that the 30 nm thickness membrane had 1.14 × 10^4^ of water flux and 3.96 × 10^4^ L m^−2^ h^−1^ bar^−1^ of acetone flux, respectively. The as-prepared cellulose membranes with sub-10 nm pores exhibited wide application in the fast removal of nanoparticles and substrates with diameters larger than 10 nm from water. In another case, Li et al. pressed a three-dimensional interconnected CNT sponge into a thin membrane for sieving nanoparticles and dye molecules from water. The prepared CNT membrane could remove 80% of CdS nanoparticles (2–4 nm), 100% of Au nanoparticles (8 nm), 100% of TiO_2_ nanoparticles (12 nm), and nearly 100% of methyl orange and rhodamine B (RhB) molecules from water, respectively [91]. The CNT membrane is not only stable at high temperature and in an acid environment, but also has filter capacities up to 45 L g^−1^. 

### 3.2. Removal of Organic Contaminants

Organic pollutants such as pesticides, hydrocarbons, phenols, oils, and pharmaceuticals can reduce the amount of dissolved oxygen in water in their oxidative decomposition process, thus endangering the aquatic organisms and damaging whole ecosystems. Karim and co-workers prepared a nanoporous membrane with cellulose nanocrystals as functional entity in chitosan via a freeze-drying process followed by compacting [16]. Although the as-prepared membrane had a low water flux (6.4 L m^−2^ h^−1^ bar^−1^), it could remove 98% of Victoria Blue 2B, 84% of Methyl Violet 2B, and 70% of Rhodamine 6G from water, respectively. The mechanism analysis indicated that this membrane adsorbed dyes mainly through electrostatic attraction and hydrogen bonds. Lee et al. developed a superhydrophobicity/superoleophilicity membrane by synthesizing vertically-aligned multi-walled carbon nanotubes (VAMWNTs) on a stainless steel mesh [92]. The contact angles of the obtained membrane for water and diesel were 163° and 0° respectively. It could efficiently separate diesel and high-viscosity lubricating oil from water. These properties make the VAMWNTs membrane very promising for the oil/water separation and oil spill cleanup. Liang and co-workers fabricated free-standing carbonaceous nanofiber (CNF) membranes by a simple casting process for water purification [93]. The obtained CNF membrane could efficiently remove methylene blue at a high flux of 1580 L m^−2^ h^−1^, and the adsorption performance cloud be easily regenerated after a simple HCl wash.

Xi and co-workers fabricated a superhydrophobic/superoleophilic NF membrane by immobilizing silver (Ag) nanoclusters on polyacrylonitrile (APAN) nanofibers [94]. The resultant APAN-Ag membrane showed a high water contact angle of 162.4° ± 1.9°, a low oil contact angle of 0°, a self-cleaning surface with water contact-angle hysteresis of 3.4° ± 0.9°, and low water-adhesion propensity. In addition, this APAN-Ag membrane could efficiently separate oil/water mixtures in both hyper-saline and various pH environments. In another study, Cao et al. prepared a highly water-selective hybrid membrane for the water/ethanol separation by incorporating g-C_3_N_4_ nanosheets into a matrix made of sodium alginate [95]. The as-prepared hybrid membrane exhibited an optimal pervaporation performance with a permeation flux of 2469 g m^−2^ h^−1^ and a separation factor of 1653 for the dehydration of 10 wt % water/ethanol mixture at 76 °C.

**Figure 6:**
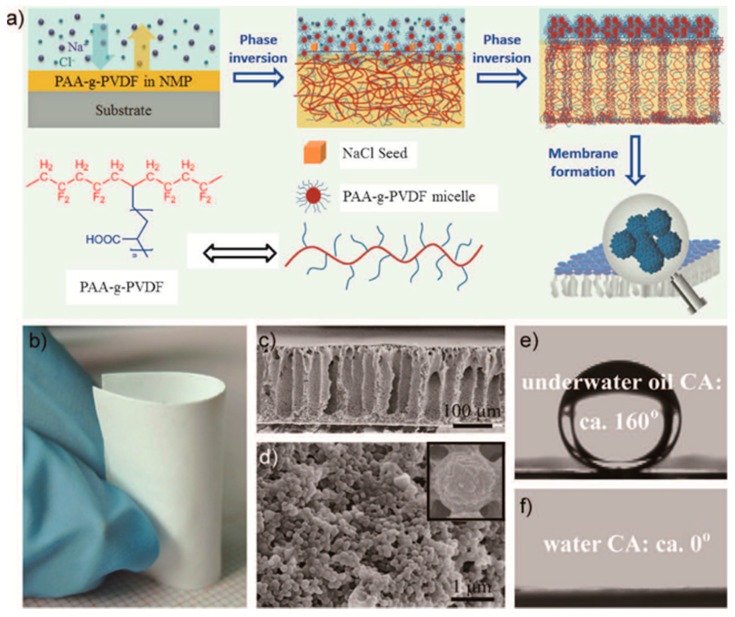
(**a**) Schematic of the preparation of PAA-g-PDVF membrane; (**b**) photograph of the as-prepared PAA-g-PDVF membrane; (**c**) cross-section and (**d**) top-view of the membrane; (**e**) image of an underwater oil droplet; (**f**) image of a water droplet on the membrane. Figures are reprinted with permission from Ref. [57]. Copyright Wiley-VCH, 2016.

Zhang et al. fabricated a superoleophobic poly(acrylic acid)-grafted PVDF (PAA-g-PVDF) membrane through a slat-induced phase inversion [57], as shown in Figure 6. The as-prepared PAA-g-PVDF could separate both surfactant-free and surfactant-stabilized oil-in-water emulsions, either under the pressure of 0.1 bar or under gravity, with high separation efficiency (˃99.99 wt % pure water) and high flux (23,200, 16,800, 15,500 L m^−2^ h^−1^ bar^−1^ of flux for hexadecane/H_2_O, toluene/H_2_O, diesel/H_2_O, respectively). Moreover, the PAA-g-PVDF membrane could be used repeatedly after a simple treatment of water washing. Obaid and co-workers developed an oil/water separation membrane by incorporation of NaOH nanoparticles inside the polysulfone (PSF) nanofibers [96]. The NaOH-modified PSF nanofiber membrane could remove almost 100% oil from water with a 5.5 m^3^ m^−2^ day water flux. In general, superhydrophobic/superoleophilic or superhydrophilic/superoleophobic nanoporous membranes can effectively separate oil/water mixtures. But for oil-water-emulsions, its separation efficiency is relatively low, and therefore surfactants are often needed to overcome this disadvantage. Moreover, a rough material surface is also beneficial for improving the separation efficiency of the nanoporous membrane.

**Figure 7:**
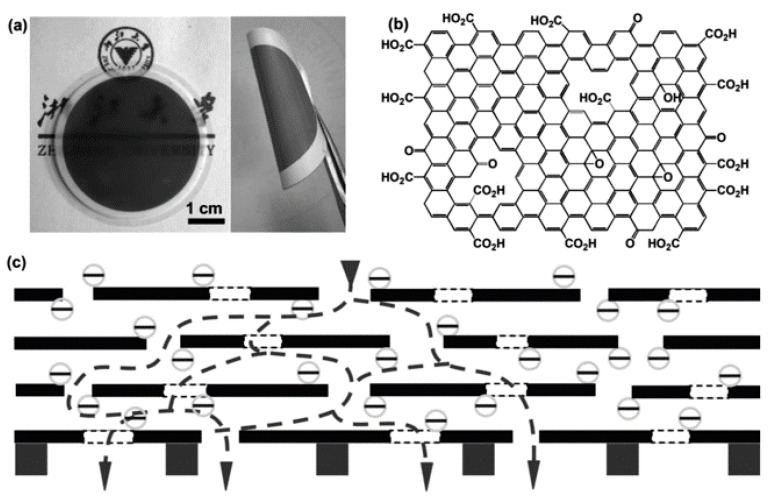
(**a**) Photographs of ultrathin graphene nanofiltration membranes (uGNM) coated on an anodic aluminum oxide (AAO) disk and a twisted uGNM coated on a PVDF membrane; (**b**) the structure of the base-washed GO; (**c**) schematic view for possible permeation route. Figures are reprinted with permission from Ref. [34]. Copyright Wiley-VCH, 2013.

Han et al. fabricated ultrathin (22–53 nm thick) graphene nanofiltration membranes (uGNM) with 2D nanochannels by a simple filtration-assisted assembly method (Figure 7) [34]. The as-prepared uGNM could sieve 99.8% of methyl blue and 99.9% of direct red 81. In addition, the water flux of uGNM reached 21.8 L m^−2^ h^−1^ bar^−1^. The water purification mechanism analysis revealed that the uGNM rejected dyes, mainly through both physical sieving and electrostatic interaction. 

GO-based nanoporous membranes have been applied widely for removing organic contaminants. For instance, Huang and co-workers prepared an integrated and continuous GO membrane on ceramic hollow fiber substrates via a vacuum suction method [97]. The obtained GO membrane could successfully separate dimethyl carbonate from water due to its preferential water sorption ability and fast water diffusivity through the GO layers. At 25 °C and 2.6 wt % feed water content, the permeate water content reached 95.3 wt % with a permeation flux of 1702 g m^−2^ h^−1^. Tang et al. fabricated free-standing GO thin membranes by using a pressurized ultrafiltration method for separation of ethanol/water mixtures [98]. The prepared GO films exhibited a layered microstructure with high structural stability and hydrophilicity. Attributed to the effect of intermolecular hydrogen bonding between water molecules and the functional groups on GO nanosheets, the fabricated GO film showed high retention for water (water/ethanol selectivity is 227) and could dehydrate 85 wt % ethanol aqueous solution at 24 °C. 

**Figure 8:**
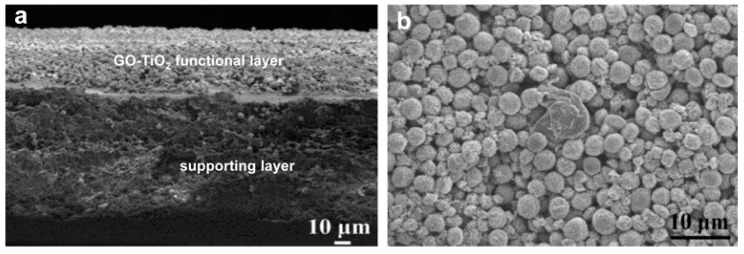
Scanning electron microscopy (SEM) images of GO-TiO_2_ membrane ((**a**) cross view; (**b**) top view). Figures are reprinted with permission from Ref. [99]. Elsevier, 2013.

Due to its excellent photocatalytic oxidation and antifouling properties, TiO_2_ nanoparticles have also been employed for organic wastewater purification. For instance, Gao and co-workers prepared a GO-TiO_2_ microsphere hierarchical membrane by assembling the GO-TiO_2_ microsphere composites on the surface of a polymer filtration membrane for concurrent water filtration and photo-degradation (Figure 8) [99]. The permeate flux of the obtained GO-TiO_2_ membrane reached 60 L m^−2^ h^−1^, which is around nine times the performance of typical commercial membranes. Moreover, under ultra-violet (UV) irradiation, TiO_2_ can be excited to generate highly oxidative species, electron holes, and hydroxyl radicals (OH·), and these species can degrade organic matter. The GO-TiO_2_ membrane could remove more than 90% of dyes (rhodamine B, acid orange 7) and humic acid (HA) from water under UV irradiation. This type of membrane has a bright future in the field of clean water production.

Besides GO, other kinds of 2D nanosheet materials can be used to fabricate nanoporous membranes for water purification. In order to reach high water flux values, Sun and co-workers prepared laminar MoS_2_ and WS_2_ membranes for the separation of Evans blue molecules in succession [100,101]. The as-prepared laminar MoS_2_ membrane exhibited 89% of rejection for Evans blue molecules and 245 L m^−2^ h^−1^ bar^−1^ of water flux. It was found that the layered WS_2_ nanosheet membrane could block over 90% of Evans blue molecules with a water flux of 730 L m^−2^ h^−1^ bar^−1^.

### 3.3. Removal of Biological Contaminants 

**Figure 9:**
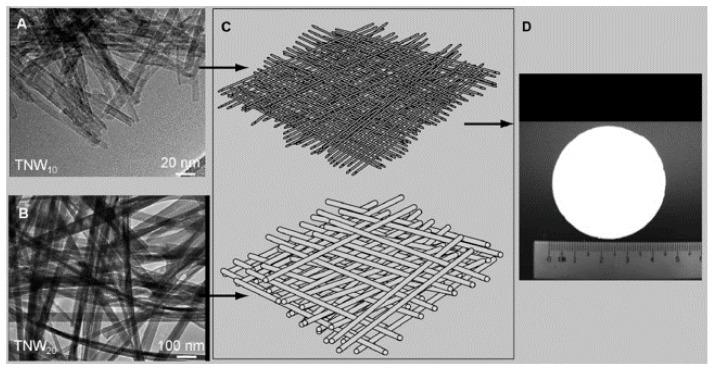
Components of the hierarchical layer of the TiO_2_ nanowire ultra-filtration (UF) membrane. (**A**) Transmission electron microscopy (TEM) image of TiO_2_ nanowires with diameter of 10 nm (TNW_10_); (**B**) TEM image of TNW_10_; (**C**) schematic profiles of the TiO_2_ nanowire UF membrane; (**D**) digital photo of the TiO_2_ nanowire UF membrane. Figures are reprinted with permission from Ref. [102]. Copyright Wiley-VCH, 2009.

Biological contaminants such as algae, planktons, bacteria, and virus are responsible for causing several illnesses that called generically waterborne diseases. Zhang and co-workers developed a TiO_2_ nanowire UF membrane with a layered hierarchical structure via an alkaline hydrothermal synthesis and hot-press process [102]. As shown in Figure 9, the TiO_2_ nanowires with diameter of 10 nm (TNW_10_) could serve as the functional layer, while TiO_2_ nanowires with a diameter of 20 nm (TNW_20_) were laid as the supporting layer. The as-prepared TiO_2_ UF membrane could successfully separate polyethylene glycol (PEG), polyethylene oxide (PEO), HA, and *E. coli* from water. It could also destroy organic pollutants (such as PEG, PEO) and inactivate biological pollutants (such as *E. coli*) under UV irradiation. Chen et al. fabricated rGO NF membrane intercalated with CNTs on porous ceramic microfiltration membranes by a facile vacuum-assisted filtration method [103]. The obtained rGO-CNT hybrid NF membrane exhibited 20–30 L m^−2^ h^−1^ bar^−1^ permeability and ˃99% retention of nanoparticles, dyes, bovine serum albumin, sugars, and HA.

**Figure 10:**
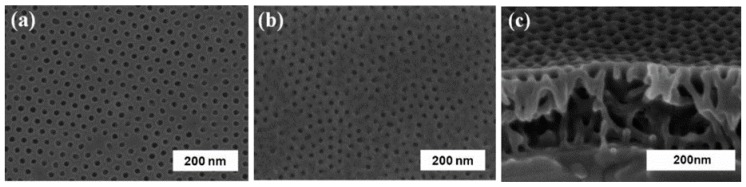
SEM images of nanoporous membrane. (**a**) top surface; (**b**) bottom surface; (**c**) cross-sectional view. Figures are reprinted with permission from Ref. [104]. Copyright Wiley-VCH, 2008.

Yang et al. developed a bilayered nanoporous membrane for virus filtration [104]. The upper layer is a nanoporous film with a pore size of ~17 nm and a thickness of ~160 nm (Figure 10), which was prepared as a polystyrene-block-poly(methyl methacrylate) copolymer. The lower layer is a conventional micro-filtration membrane, which serves to enhance mechanical strength. The as-prepared membrane exhibited high selectivity towards human rhinovirus type 14, and could sieve nearly 100% of human rhinovirus type 14 from phosphate buffer solution. In addition, this membrane showed good stability even at a pressure of 2 bar. Sato et al. fabricated a nanofibrous composite membrane via infusing surface-modified ultra-fine cellulose nanofibers (diameter: 5–10 nm) into an electrospun nanofibrous scaffold [105]. The water flux of the created membrane could reach 85 L m^−2^ h^−1^ bar^−1^, and this membrane could simultaneously remove 99.99% of MS_2_ bacteriophage virus and 99.9999% of *E. coli*. The mechanism analysis indicated that the nanofibrous composite membrane mainly sieved bacteria because of its pore size, and it adsorbed viruses (negatively charged) through electrostatic attraction.

### 3.4. Summary of the Fabrication and Water Purification Performances of Membranes

In summary, the above-mentioned examples of nanoporous membrane for three types of water pollutants are listed in Table 1. As can be seen this Table, phase inversion is the most commonly used method to prepare various inorganic membranes, and both phase inversion and electrospinning strategies have been usually employed for fabricating organic- and inorganic-organic hybrid membranes. In addition, organic membranes showed commonly a higher water flux, but a lower removal rate compared to inorganic membranes. In contrast, inorganic membranes have a higher removal rate and selectivity towards target pollutes, but a lower water flux when compared with organic membranes. However, inorganic-organic hybrid membranes combine the advantages of both types of membranes, and not only maintained high water flux but also showed high selectivity and removal rate for target pollutants. Therefore, nanoporous inorganic-organic hybrid membranes have attracted wide attention regarding their applications in water purification. Moreover, regarding the filtration mechanisms, it can be found that all three types of membranes (organic, inorganic, and inorganic-organic hybrid) are able to separate the target water pollutants via pore-size selection and electrostatic forces.

## 4. Conclusions and Perspectives

In this review, we have summarized and discussed various fabrication strategies of nanoporous membranes and their applications in the field of water purification. We have found that the solute and water permeability play important roles in the membrane performance. The membranes separate pollutants (such as inorganic ions, organic molecules, nanoparticles, viruses, etc.) from water mainly through size exclusion and solution diffusion. Although these reported membranes are demonstrated successfully at the laboratory scale, upscaling them to low-cost, industrial-scale modules is still a big challenge. To overcome the barrier toward successful upscaling and commercialization will need a combined, collaborative effort by research institutions and industrial companies. 

In our opinion, the development direction of the next-generation of thin membranes for water purification may include the following aspects. First, it is important to improve the selectivity of desalination membranes. Enhanced membrane selectivity not only can improve the quality of water but can also eliminate the need for additional purification stages. For example, the selective separation of boric acid from seawater can reduce both energy usage and the cost of desalination [17]. The use of molecular-level design approaches to prepare thin, defect-free, and selective layer on microporous supports may be a good way to achieve this aim. Second, it is necessary to improve the fouling resistance of nanoporous membrane. Surface grafting of fouling-resistant polymer such as zwitterionic polymers and PEO may be the potential strategies for the next-generation membranes [106,107]. Surface grafting, however, will not impart fouling resistance to the interior of the pore walls. Thence, the choice of other suitable anti-fouling modifiers embedded in the membrane matrix will be crucial. Third, it is very important to overcome the high sensitivity of the current polyamide membranes to oxidants such as chlorine and ozone. Fourth, the fabrication of multi-functional membranes is a research field with high innovation and potential, which will lead to the production of membranes with separation, catalytic, degradation, anti-fouling, antibacterial, and other functions [25,108,109]. Although there are still many challenges to be overcome for the industrial production of low-cost and efficient nanoporous membranes for water purification, we believe that it will be realized in the near future under the joint efforts of scientists, industrial engineers, governments, and investors.

## Figures and Tables

**Table 1:** Application examples of nanoporous membranes for water purification ^a^.

Membrane	Synthesis Method	Target and Efficiency	Water Flux	Ref.
PVDF	Electrospinning	NaCl (<280 ppm)	5–28 Kg m^−2^ h^−1^	[79]
Aquaporin reconstituted	Vacuum suction and amine-catrchol adduct formation	NaCl (66.2%), MgCl_2_ (88.1%)		[81]
Zr-MOF	Solvothermal synthesis	Al^3+^ (99.3%), Mg^2+^ (98.0%), Ca^2+^ (86.3%)	0.28 L m^−2^ h^−1^ bar^−1^	[83]
CNT-PcH	Electrospinning	NaCl (˃99.99%)	24–29 L m^−2^ h^−1^	[82]
MCM41-PA-TFN	Interfacial polymerization	NaCl (97.9% ± 0.3%), Na_2_SO_4_ (98.5% ± 0.2%)	46.6 ± 1.1 L m^−2^ h^−1^	[68]
GO-PA-TFN	Interfacial polymerization	NaCl (93.8% ± 0.6%), Na_2_SO_4_ (97.3% ± 0.3%)	59.4 ± 0.4 L m^−2^ h^−1^	[69]
K^+^-controlled GO	Drop-casting	Mg^2+^, Ca^2+^, Na^+^ (~100%)	0.36 L m^−2^ h^−1^	[84]
Sigle-layer graphene	oxygen plasma etching	K^+^, Na^+^, Li^+^, Cl^−^ (~100%)	10^6^ g m^−2^ s^−1^	[20]
Ti_3_C_2_T_x_ Mxene	Electrospinning	metal cations and dye cations (diameter ≥ 6 Å)	37.4 L m^−2^ h^−1^ bar^−1^	[80]
GO	Impregnation	Salt cations (6–46%), Methylene blue (46–66%), Raodamine-WT (93–95%)	27.6 L m^−2^ h^−1^ bar^−1^	[85]
GO@PAN	Vacuum suction	Na_2_SO_4_ (56.7%), Congo red	8.2 L m^−2^ h^−1^ bar^−1^	[86]
rGO	hydriodic acid vapor, water-assisted delamination	Cu^2+^, Na^+^, orange 7 (~100%)	12.0 L m^−2^ h^−1^ bar^−1^	[87]
GO-based	Shear-induced alignment	organic probe molecules (˃90%), salt cations (30–40%)	71 ± 5 L m^−2^ h^−1^ bar^−1^	[88]
bicontinous cubic	Self-assembly	${\mathrm{Br}}^{-}$ (83%), ${\mathrm{Cl}}^{-}$ (59%), ${\mathrm{SO}}_{4}^{2-}$ (33%), ${\mathrm{NO}}_{3}^{-}$ (81%)	2.8–5.7 L m^−2^ h^−1^ bar^−1^	[89]
NPN	Track-etching	Au nanoparticles (˃80%)		[71]
Cellulose	Freeze-extraction technique	Nanoparticles with diameter ˃10 nm	1.14 × 10^4^ L m^−2^ h^−1^ bar^−1^	[21]
CNT	Chemical vapor deposition	CdS (80%), Au (100%), TiO_2_ (100%) nanoparticles		[91]
CNCs	Freeze-drying process	Victoria Blue 2B (98%), Methyl Violet 2B (84%), Rhodamine 6G (70%)	6.4 L m^−2^ h^−1^ bar^−1^	[16]
VAMWNTs	Chemical vapor deposition	Lubricating oil	1580 L m^−2^ h^−1^	[92]
Ag-APAN	Electroless plating, surface modification	1,2-dibromoethane		[94]
CNs-SA	Thermal oxidation etching	Ethanol	2469 g m^−2^ h^−1^	[95]
PAA-g-PVDF	Phase inversion	hexadecane, toluene, diesel (˃99.99%)	15,500–23,200 L m^−2^ h^−1^ bar^−1^	[57]
PSF nanofibers	Electrospinning, interfacial polymerization	Soybean oil (~100%)	5.5 m^3^ m^−2^ day	[96]
uGNM	filtration-assisted assembly	99.8% of methyl blue and 99.9% of direct red 81	21.8 L m^−2^ h^−1^ bar^−1^	[34]
GO	Vacuum suction	dimethyl carbonate (95.2%)	1702 g m^−2^ h^−1^	[97]
GO	Pressurized ultrafiltration	Ethanol (~100%)		[98]
GO-TiO_2_	Self-assembly	rhodamine B, acid orange 7, humic acid (˃90%)	60 L m^−2^ h^−1^	[99]
MoS_2_	Vacuum filtration	Evans blue (89%)	245 L m^−2^ h^−1^ bar^−1^	[100]
WS_2_	Vacuum filtration	Evans blue (˃90%)	730 L m^−2^ h^−1^ bar^−1^	[101]
TiO_2_ nanowire	Hydrothermal synthesis, hot-press process	polyethylene glycol, polyethylene oxide, HA, *E. coli*		[21]
rGO-CNT	Vacuum-assisted filtration	nanoparticles, dyes, BSA, sugars, and humic acid (˃99%)	20–30 L m^−2^ h^−1^ bar^−1^	[103]
PMMA	Ultraviolet irradiation, acid rinsing	human rhinovirus type 14 (~100%)		[104]
MCCNs-PEI	Electrospinning	MS2 bacteriophage virus (99.99%), *E. coli* (99.9999%)	85 L m^−2^ h^−1^ bar^−1^	[105]

^a^ PVDF: polyvinylidene fluoride; Zr-MOF: zirconium(IV)-based metal-organic framework membrane; CNT-PcH: carbon nanotube incorporated polyvinylidene fluoride-co-hexafluoropropylene nanofiber membrane; MCM41-PA-TFN: MCM-41 silica nanoparticles enhanced polyamide thin-film nanocomposite membrane; GO: graphene oxide; GO-PA-TFN: graphene oxide enhanced polyamide thin-film nanocomposite membrane; PAN: polyacrylonitrile; rGO: reduced graphene oxide; NPN: nanoporous silicon nitride; CNT: carbon nanotube; CNCs: cellulose nanocrystals; VAMWNTs: vertically-aligned multi-walled carbon nanotubes; APAN: polyacrylonitrile; CNs-SA: g-C_3_N_4_ nanosheets incorporated into sodium alginate matrix; PAA-g-PVDF: poly(acrylic acid)-grafted PVDF; PSF: polysulfone; uGNM: ultrathin graphene nanofiltration membrane; PMMA: polystyrene-block-poly(methyl methacrylate); MCCNs: microcrystalline cellulose nanofibers; PEI: polyethylenimine.
